# Clinical utility and tolerability of JSPH-1 wireless esophageal pH monitoring system

**DOI:** 10.1186/1471-230X-13-10

**Published:** 2013-01-15

**Authors:** Jun-Nan Li, Chun-Lun Liu, Xiao-Hong Tao

**Affiliations:** 1 Department of Gastroenterology, The First Affiliated Hospital of Chongqing Medical University, Chongqing 404100, China

**Keywords:** Esophagus, Abnormal acid exposure, Esophageal pH monitoring, Wireless pH monitoring system, Gastroesophageal reflux disease

## Abstract

**Background:**

Wireless esophageal pH monitoring system is an important approach for diagnosis of gastroesophageal reflux disease (GERD), the aim of this study is to test the tolerability and utility of the first wireless esophageal pH monitoring system made in China, and evaluate whether it is feasible for clinical application to diagnose GERD.

**Methods:**

Thirty patients from Department of Gastroenterology of The First Affiliated Hospital of Chongqing Medical University who were suspected GERD underwent JSPH-1 pH capsule. The capsule was placed 5 cm proximal to the squamocolumnar junction (SCJ) by endoscopic determination, the data was recorded consecutively for 48 hours. Then all pH data was downloaded to a computer for analysis. The discomforts reported by patients were recorded.

**Results:**

30 patients were placed JSPH-1 pH capsule successfully and completed 24-hour data recording, 29 patients completed 48-hour data recording. One patient complained of chest pain and required endoscopic removal. No complications and interference of daily activities were reported during data monitoring or follow-up period. 48-hour pH monitoring detected 15 patients of abnormal acid exposure, on day1 detected 9 patients, the difference had statistical significance (*P*<0.01). Positive symptom index (SI) was identified in 3 patients with normal pH data in both 24-hours. In total, 48-hour monitoring increased diagnosis of GERD in 9 patients.

**Conclusion:**

48-hour esophageal pH monitoring with JSPH-1 wireless pH monitoring system is safe, well tolerated and effective. It can be feasible for clinical application to diagnose GERD.

## Background

In western countries, the prevalence of GERD is approximate 10%-20% [1] and has the trend of ascendant. In recent decades, several population-based studies have shown that the prevalence of GERD has significantly increased in Asia [2]. GERD has gradually become one of the most common upper gastrointestinal disorders. Patients are suffering from acid reflux, life and health are seriously impacted. So the diagnosis of GERD seems more and more important. Currently, the main approaches for diagnosis of GERD include endoscopy, symptom questionnaire (such as gastroesophageal reflux disease questionnaire), diagnostic testing with proton pump inhibitor (PPI), gastroesophageal reflux monitoring (esophageal pH monitoring, esophageal impedance monitoring, esophageal pressure monitoring, high-frequency endo-luminal ultrasound, esophageal bilirubin reflux monitoring) and so on. Esophageal pH monitoring serves an important role in numerous methods, because it can quantify esophageal acid exposure and provide objective pH data for diagnosis. Ambulatory esophageal pH monitoring involves an electrode in a catheter, and can transnasally into the esophagus, but the catheter causes pharyngeal, nasal, oral discomfort, and social embarrassment. Consequently, patients would like to keep more sedentary during monitoring period, and modify normal activities/or diet which may provoke acid reflux [3,4]. In addition, movement of catheter can lead electrode off the primary position. All of this can result in inaccurate pH data and final diagnosis.

With Technologic advances, wireless pH monitoring system has been introduced into clinical application. The Medtronic Bravo pH monitoring system was the United States Food and Drug Administration class I approved wireless pH monitoring system [5], this system includes a radiotelemetry pH capsule attached to the mucosal wall of esophagus, which simultaneously measures pH (frequency is 6 s) and transmits (frequency is 75 s) data to a wireless data receiver, that can decrease discomfort and social inconvenience, patients could better tolerate and keep normal daily activities easily [5,6]. Moreover, wireless pH capsule can decrease false-negative results. Many authors have confirmed the clinical utility and tolerability of wireless pH monitoring system [7-10]. Recently, a study also showed wireless pH capsule provided new information altering patient management and changing patient diagnoses [11].

JSPH-1 wireless pH monitoring system is designed by Jinshan Science and Technology Inc. of Chongqing in China. With examination and approval of Ethical Committee of the First Affiliated Hospital of Chongqing Medical University in China, we performed a clinical test to confirm the sWith examination and approval of Ethical Committee of the First Affiliated Hospital of Chongqing Medical University in China, we performed a clinical test to confirm the safety, tolerance, and clinical utility of this new wireless pH monitoring system and to evaluate whether it is feasible for clinical application.afety, tolerance, and clinical utility of this new wireless pH monitoring system and to evaluate whether it is feasible for clinical application.

## Methods

### JSPH-1pH capsule system

**Figure 1:**
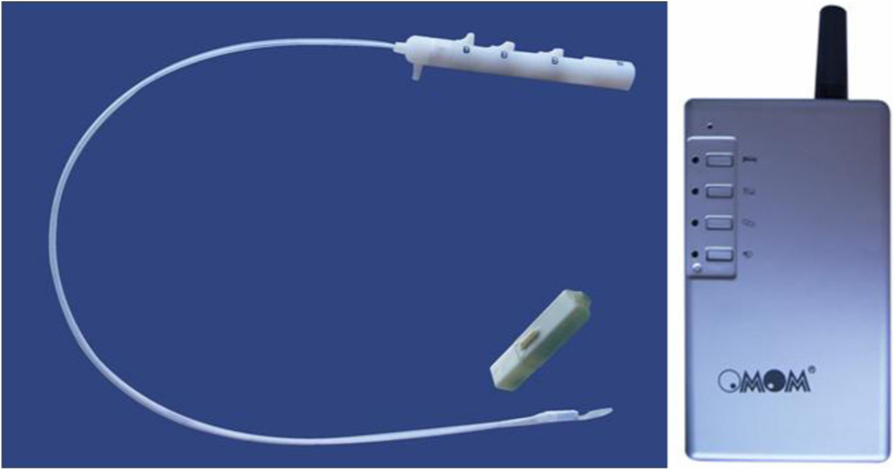
(Color graph) JSPH-1pH capsule system: delivery system, capsule and wireless receiver.

**Figure 2:**
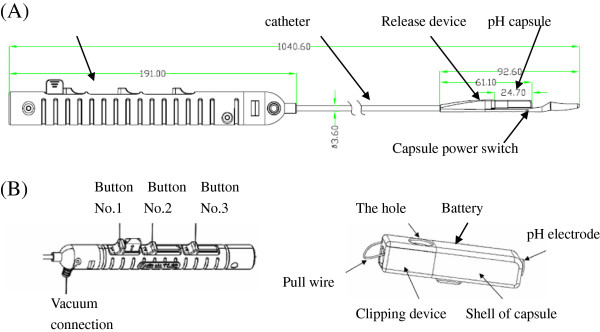
(A)Prepackaged device including both a delivery system and a capsule, unit of length is millimeter (mm). (B) Separated handle of the delivery system and JSPH-1 pH capsule for detail introduction.

Materials of the JSPH-1 pH wireless monitoring system include a capsule, a delivery device and a wireless data receiver (Figure 1), a capsule by the size of 26.5 mm×6.0 mm×5.5 mm, the container constructed with acid–resistant and alkali–resistant material, containing an antimony pH electrode, a silver oxide internal battery, a radio transmitter and a clipping device (Figure 2). The antimony pH electrode is hypersensitive to acid, but it also can respond to alkaline. The pH sensor detects pH data every 3 s and transmits to wireless receiver by the radio transmitter every 60s, and the silver oxide battery enables 96-hour or longer pH-metry. In proximal of the capsule, there is a hole with an opening electrical insulating clip. When the clip closed, capsule would be fixed on esophageal mucosa wall.

Three movable buttons marked 1, 2, 3 are lined on the handle of delivery device (Figure 2). Firstly, sliding the button marked number 1 to the end of proximal rail can close the clip. Then, sliding the button marked number 2 can release the clip from delivery device. At last, sliding the button marked number 3 releases the whole capsule from the delivery device. The three buttons must be slid in turn to avoid laceration of esophageal mucosa, severe ache or huge hemorrhage and ensure a safe process of capsule placement.

### Patients screening

With examination and approval of Ethical Committee of The First Affiliated Hospital of Chongqing Medical University in China, we performed a prospective clinical test. All patients gave their written informed consent to participate and the study was conducted in accordance with the Declaration of Helsinki. Patients came from Department of Gastroenterology of First Affiliated Hospital of Chongqing Medical University from April 2011 to August 2011, aged 18 to 65 years, with typical symptoms (heartburn, regurgitation) and/or atypical symptoms (chest pain, asthma and chronic cough with no reason, chronic pharyngitis) of GERD, or PPI therapy test positive were fit for inclusion. Exclusion criteria of this study were: pregnant women, with history of bleeding tendency or coagulation function abnormal, proximal esophagus or nasopharynx suspect obstruction, significant erosive of esophageal mucosa, a history of upper gastrointestinal surgery within 6 months, gastroenteric obstruction, uncooperative patients, or hypersensitivity to the material. All patients provided basic information and disease history, and were requested to be off antacids for 24 hours as well as histamine-2 receptor antagonists for at least 5 days. PPI should be stopped for 7 days. Fasting state with nothing taking should continue at least 6 hours prior to the study. All patients signed informed consents file before the test.

### Protocol taking

**Figure 3:**
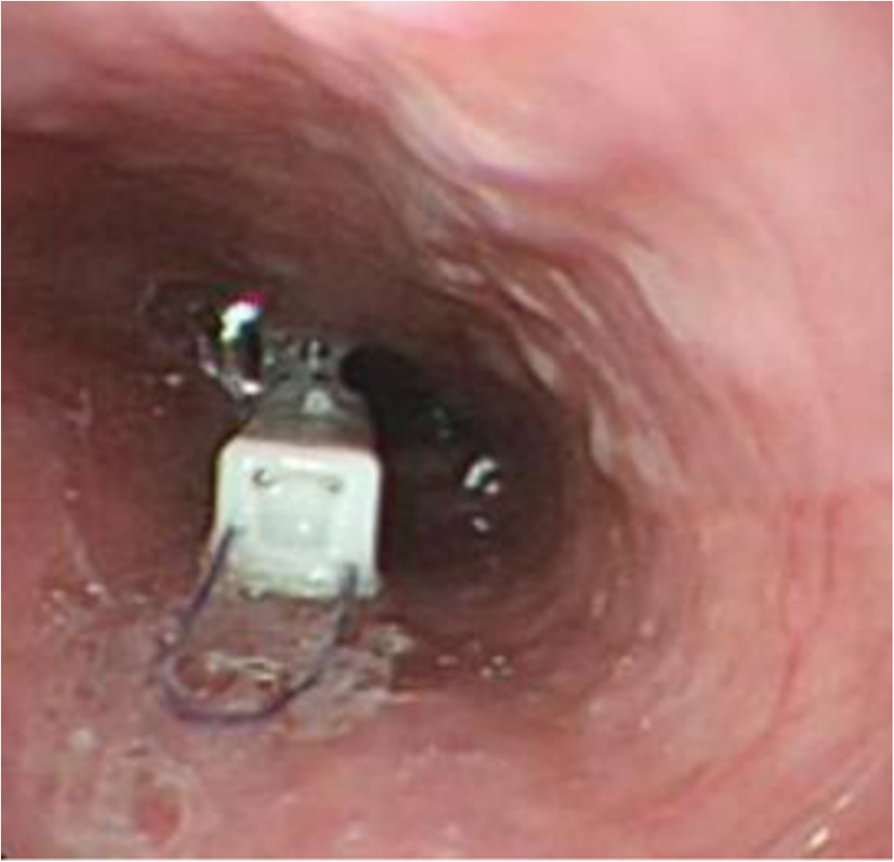
Capsule was placed 5-cm above SCJ and was confirmed by endoscopy.

Before placement, pH capsule was put into the buffer solution with different pH (1.07, 4.00 and 7.01) for calibration, this procedure was finished in Motility Laboratory. Then patients were taken to endoscopic room, underwent endoscopy to measure the accurate position of SCJ and detect esophageal lesions. All patients were kept conscious without sedation in the whole process. When JSPH-1 capsule reached posterior wall of orpharynx, patients coordinated swallowing act to assist the capsule enter into esophagus. Paid attention to the scales on the delivery catheter when the catheter slowly transmitted into esophagus, make sure the pH capsule was placed 5 cm above SCJ (Figure 3). Subsequently, connected vacuum pump to the handle, turned on it to apply suction pressure stabilized at 550 mmHg for 5 s, the adjacent esophageal mucosa was immediately drawn in. Sliding the movable button marked number 1 to the end of proximal rail to close the clip around the drawn mucosa immediately, thus attaching the capsule to esophageal mucosa wall. The button marked number 2 was slid to release the clip from the delivery catheter, the button number 3 release the capsule from the delivery system. Finally, turned off the vacuum pump, removed it from the handle and took out the delivery catheter slowly. The attached capsule would be checked directly by endoscopy. Patients were told to take the wireless data receiver in the pockets or bags, with the distance no more than 2 meters at any time during recording period. Patients were encouraged to keep normal daily activities except for alcohol or carbonic acid beverage drinking. The drugs which influenced acid reflux were not permitted. Instructions about keeping detail dairy were performed, dairy including daily activities, food taking, reflux symptoms(chest discomfort, acid reflux, heart burn)and other events which would be useful in the later interpretation of pH data. Patients were informed to return 24 hours latter, at that time the data would be downloaded to a computer for primary analysis. All patients underwent chest X-ray to document capsule attachment, if pH capsule was detached, the patients would exit the later study. Others took back the diary for continue recording in another 24 hours. 48 hours later, all patients turned in receivers and diaries. All sufficient data was downloaded to a computer and automatically analyzed by Jin Shan pH monitoring working station. All pH data was provided along with pH tracings of day1,day2 and the 48-h test period. Follow-up time would last for 3 months after capsule detachment.

### Data analysis

**Figure 4:**
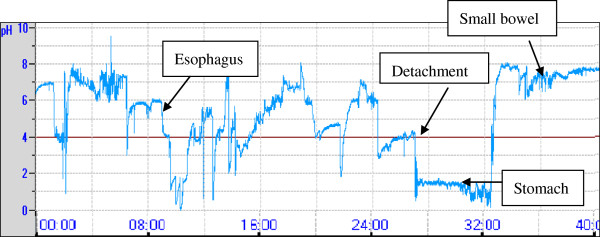
**Premature detachment of JSPH-1 pH capsule in our study (n=1). **The sudden prolonged dropped in pH represented pH capsule detached into stomach, and then sharp raised when the capsule entered the small intestine. The data following capsule detached were invalid.

The pH data recording time continues at least 24 hours was available, less than 24 hours considered insufficient. If the pH data sudden dropped to below 2 (pH < 2) for more than 2 hours (in stomach), then pH data rise to normal, no longer fall in remain trial (in small bowel). In this phenomenon, the capsule was considered detached, if it was confirmed by X ray, the data was deemed invalid (Figure 4).

Johnson-DeMeester score [12,13] was used to evaluate abnormal acid exposure, the score greater than 14.7 defined abnormal acid exposure positive [14]. SI was used to express symptom-reflux associations. The values more than 50% were considered positive [15,16]. The software of SAS 9.2 was used to carry out all the analysis needed in our study.

## Results

Originally, thirty-two patients were preliminary screened. One patient requested to exit due to distant residence. Another one was excluded because of severe arrhythmia. The rest of the 30 patients were finally selected (Table 1), 19 female (63.3%), 11male (37.7%), median of age 47.0. The attachment confirmed by endoscopy was successful in all patients.

**Table 1:** Patient characteristics

**Number of patients**	**(n, %)**
Total number	30
Sex	
Male	19(59.38%)
Female	11(40.62%)
Medications	
On	7(23.33%)
Off	23(76.67%)
Primary symptoms	
Heartburn	18(60.00%)
Regurgitation	5 (16.67%)
Atypical symptoms	16(33.33%)
(Chest pain or abdominal distention)	

During placement process, 26 patients had mild discomfort with slight nausea. Eighteen patients (60%) had symptoms related to capsule attachment, no patient had nausea. Symptoms related to capsule attachment were: foreign body sense in 12(40%), chest pain in 10(33%), dysphagia in 8(27%), and 5(17%) had more than one symptom. Those symptoms disappeared when capsule was dislodged (Table 2).

**Table 2:** Severity of symptoms related to attachment of JSPH-1

	**Chest pain**	**Foreign Body**	**Dysphagia**
	(n, %)	Sensation (n, %)	(n, %)
Mild	8(80%)	7(58%)	5(63%)
Moderate	1(10%)	5(42%)	3(27%)
Severe	1(10%)	0	0
Very severe	0	0	0

*n represents the number of patients.

**Figure 5:**
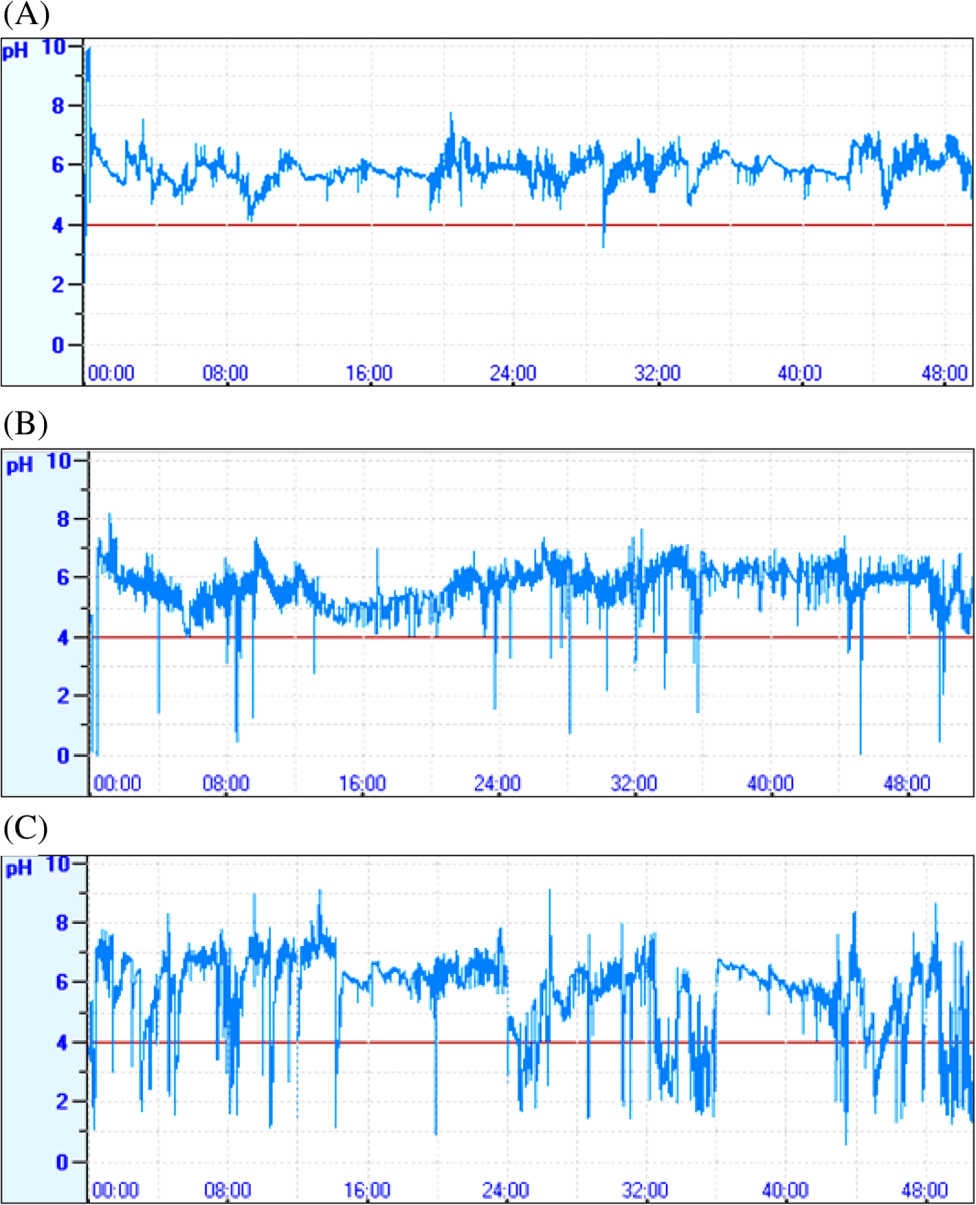
Correct capsule position in patients, forty-eight-hour pH tracings using Jinshan pH monitoring working station. (A) A normal subject. (B) A GERD patient with slight reflux. (C) A GERD patient with serious reflux.

We detected capsules detachment by pH data or documented by X-ray. In 28 patients (93.3%), capsules dislodged within 4 to 12 days, 1 patient at 27th hour and 1 patient almost at 25th day. Capsules spontaneously dislodged in 29 patients. 1 patient complained of chest pain, and we performed endoscopic removal on 7th day according to her requirement. No one complained of discomfort or limitation of daily life during detachment and excretion of the capsule or in follow-up period (Figure 5).

Effective pH monitoring extended 48 hours in 29 patients (96.7%). Abnormal acid exposure occurred in 9 patients (30.0%) on day1, 12 patients (41.4%) on day2, and 15 patients (51.7%) in extended 48-h. the difference of acid exposure had statistical significance between 48-h and day1 (P=6.997E-04), also between 48-h and day2 (P=1.052E-05) (Table 3).

**Table 3:** The result of abnormal acid exposure in 48-h, day1 and day2

	**24-h (n, %)**	**48-h (n, %)**	***P***
day1	9(31.0%)	15(65.5%)	6.997E-04
day2	12(37.9%)	15(65.5%)	1.052E-05

*n represents the number of patients with abnormal acid exposure positive.

In total, 26 patients reported symptoms of GERD. SI was positive in 9 patients (31.0%) in day1, 11 patients (37.9%) day2, 19 patients (65.5%) in 48-h.The difference of SI had statistical significance between the 48-h and day1 (*P*=0.0114), but no statistical significance between 48-h and day 2 (*P*=0.2344) (Table 4).

**Table 4:** The result of SI in 48-h, day1 and day2

	**24-h (n, %)**	**48-h (n, %)**	***P***
day1	9(31.0%)	19(65.5%)	0.0114
day2	11(37.9%)	19(65.5%)	0.2344

*n represents the number of patients with positive SI.

Six patients had been identified during 48-h with abnormal pH data but not been detected on day1. In 14 patients (46.7%) with normal pH data on both day1 and day2, positive SI was identified in 3 patients (21.4%). therefore, 48-h extended monitoring increased 9 patients (31.0%) diagnosis of GERD.

## Discussion

As far as our knowledge goes, this is the first clinical test based on a new wireless pH monitoring system made in China. The JSPH-1 pH capsule was designed with a high monitoring frequency of 3 s, transmission frequency of 60s, working hour extends to 96 hours, and pH value can be detected from 0 to 9, the measure accuracy reach±0.5. That may be advanced when evaluate sensitivity and diagnostic yield in patients with an indeterminate result for GERD after 48-h or 24-h pH monitoring [17,18]. Because of high working efficiency and long working hours, JSPH-1 new pH monitoring system can detect extremely slight abnormal esophageal acid exposure, and observe clinical effect of anti-acid reflux medicine. It is still unknown whether JSPH-1 pH capsule is more effective than BRAVO pH capsule system in clinical complication.

Foreign body sensation in this study was the most common symptom reported by patients, similar results were showed in some studies based on Bravo pH capsule system [5,19]. Endoscopic removal should be performed when patients have serious symptoms for more than 48 hours [20] or capsule was still attached for more than two weeks. A cold snare would be used to loosely around the base of the attachment area between the capsule and the esophageal mucosa, then gently pulled the snare to-and-fro, if the capsule fails to detached, closing the snare, with the position as close to the base of capsule as possible, and then the hot snare mode should be promoted to cut the attachment mucosa successfully. In our study, 1 patient (female) reported chest pain during data monitoring with depressive and nervous emotion. The pathological mechanism of chest pain might be connected to hypertensive esophageal contractions caused by intraesophageal capsule [21]. But in our study, depressive emotion might be also an important factor.

Many studies based on BRAVO pH capsule system had showed extended 48-h pH monitoring increased diagnosis of GERD [22,23]. A retrospective study [19] with 100 patients performed BRAVO pH capsule system confirmed 48-h extended monitoring detected 43.4% more patients with abnormal esophageal acid exposure. With JSPH-1 pH capsule, extended 48-h pH monitoring also seems more sensitive than 24-h monitoring because 48-h pH monitoring increased 6 patients (21.7%) detection of abnormal esophageal acid exposure when compared to day1, and increased 3 patients (10.3%) detection when compared to day 2. If monitoring time was 24-h or analysis only 24-h pH data, these patients would have been misdiagnosed. Positive SI was identified in 3 patients out of 14 patients with normal pH data, According to Rome III criteria, these patients should be included in GERD disease [24,25]. In total, extended 48-h pH monitoring increased 9 patients (31.0%) diagnosis of GERD when 24-h pH monitoring data could not make diagnosis [19], but the increased rate was lower than the result with BRAVO pH capsule system. Some study demonstrated 48-hour pH monitoring increases the risk of false positive studies when the capsule is prematurely dislodged [26], in our study, the capsule prematurely dislodged at 27th hour occurred in 1 patient, the data was excluded on day 2 to avoid the false positive.

48-h pH monitoring detect positive SI more than day1, the difference had statistical significance. Therefore, 48-h extended monitoring could increase diagnosis of GERD according to abnormal pH data and SI.

Wireless pH monitoring systems are favorable for diagnosis of GERD, but also have common disadvantages [25]: proximal esophageal reflux monitoring is not feasible, non-acid reflux can not be evaluated. Multichannel intraluminal impedance-pH (MII-pH) testing [27,28] is a relatively new technique, it can detect non-acidic reflux episodes through changes of impedance in esophageal lumen, especially air reflux episodes when pH data is normal. If JSPH-1 pH capsule system introduces an impedance electrode in container on the basis of fitful or smaller measure of capsule which would be a great improvement in diagnostic field of GERD.

Although our study had been designed carefully, several limitations to this study should be mentioned. Firstly, all of the subjects were endoscopy normal patients with acid reflux symptoms, the major pathmechanism related with esophageal mucosal hypersensitivity, the discomfort of pH capsule attachment in monitoring time might be amplified. Secondly, the study was not designed to compare with other wireless systems directly. It is still unknown whether JSPH-1 pH capsule is more effective than other wireless systems in clinical complication or not. Thirdly, our study was a small sample clinical test carried out in a single center, the data could provide valid information but the result might be with fairly error, thus, a large and multiple centers with prospective study with JSPH-1 pH capsule will be needed in the future.

## Conclusions

JSPH-1 pH capsule system is a new wireless esophageal pH monitoring system with high working efficiency, high accuracy and long working hour. In this clinical test, patients underwent JSPH-1 pH capsule have a well tolerance in the whole procedure, no interference in their daily activities. 48-h extended pH monitoring increased in diagnostic field of GERD. Consequently, JSPH-1 pH capsule system is safe, well tolerant and effective. It can be feasible for clinical application to diagnose GERD.

## Competing interests

All authors have no financial competing interests with each other, or other people. The JSPH-1 pH capsule system in this research was provided by Jinshan Science and Technology Inc. All authors have no financial competing interests with Jinshan Science and Technology Inc.

## Authors’ contributions

Li JN, Liu CL and Tao XH involved with concept and design of the study, Li JN and Liu CL performed the research and acquired the data, Li JN completed statistical analysis and drafted of the manuscript, Liu CL and Tao XH involved with critical revision of the manuscript for important content of the manuscript. All authors read and approved the final manuscript.

## Authors’ information

Jun-Nan Li, MD, Dr. of department of Gastroenterology, The First Affiliated Hospital of Chongqing Medical University.

Chun-Lun Liu, MD and PhD,associate professor of department of Gastroenterology, The First Affiliated Hospital of Chongqing Medical University, involved in clinical work and research for about twenty years.

Xiao-Hong Tao, MD and PhD,professor, Chief of department of Gastroenterology, The First Affiliated Hospital of Chongqing Medical University, involved in clinical work and research for more than thirty years, also, worked in USA for three years, specialism in operation of endoscope.

## Pre-publication history

The pre-publication history for this paper can be accessed here:

http://www.biomedcentral.com/1471-230X/13/10/prepub
